# rRNA Pseudogenes in Filamentous Ascomycetes as Revealed by Genome Data

**DOI:** 10.1534/g3.117.044016

**Published:** 2017-06-21

**Authors:** Yi Li, Rui-Heng Yang, Lan Jiang, Xiao-Di Hu, Zu-Jian Wu, Yi-Jian Yao

**Affiliations:** * Fujian Province Key Laboratory of Plant Virology, Institute of Plant Virology, Fujian Agricultural and Forestry University, 350002 Fuzhou, China; † State Key Laboratory of Mycology, Institute of Microbiology, Chinese Academy of Sciences, 100101 Beijing, China; ‡ University of Chinese Academy of Sciences, 100049 Beijing, China

**Keywords:** concerted evolution, genome sequencing, RIP, phylogeny, fungi

## Abstract

The nuclear ribosomal DNA (rDNA) is considered as a paradigm of concerted evolution. Components of the rDNA tandem repeats (45S) are widely used in phylogenetic studies of different organisms and the internal transcribed spacer (ITS) region was recently selected as a fungal DNA bar code. However, rRNA pseudogenes, as one kind of escape from concerted evolution, were reported in a wide range of organisms, especially in plants and animals. Moreover, large numbers of 5S rRNA pseudogenes were identified in several filamentous ascomycetes. To study whether rDNA evolves in a strict concerted manner and test whether rRNA pseudogenes exist in more species of ascomycetes, intragenomic rDNA polymorphisms were analyzed using whole genome sequences. Divergent rDNA paralogs were found to coexist within a single genome in seven filamentous ascomycetes examined. A great number of paralogs were identified as pseudogenes according to the mutation and secondary structure analyses. Phylogenetic analyses of the three rRNA coding regions of the 45S rDNA repeats, *i.e.*, 18S, 5.8S, and 28S, revealed an interspecies clustering pattern of those different rDNA paralogs. The identified rRNA pseudogenic sequences were validated using specific primers designed. Mutation analyses revealed that the repeat-induced point (RIP) mutation was probably responsible for the formation of those rRNA pseudogenes.

The nuclear ribosomal DNA (rDNA) is organized into two distinct multigene families in most eukaryotes, comprising the 45S and 5S rDNA repeats. The 45S rDNA repeats consist of three rRNA coding genes (18S, 5.8S, and 28S), two internal transcribed spacers (ITS1 and ITS2), and a large intergenic spacer (IGS); while the 5S rDNA repeats include the 5S rRNA transcribing region and a nontranscribed spacer (Long and Dawid 1980). The copy number of the 45S and 5S rDNA repeats varies in fungi; usually from 12 to several hundred for the 45S, and 50 to 200 for the 5S (Long and Dawid 1980; Rooney and Ward 2005; Ganley and Kobayashi 2007). The 45S rDNA repeats are arranged either in a single large tandem array or in multiple tandem arrays on one or a few chromosomes (Rooney and Ward 2005), while the 5S rDNA repeats show a more complicated arrangement and can be either dispersed throughout the genome, arranged in distinct tandem arrays, or linked to other tandemly repeated gene families (Selker *et al.* 1981; Mao *et al.* 1982; Drouin and de Sá 1995).

The predominant paradigm of multigene family evolution prior to the 1970s was known as “divergent evolution,” in which duplicated genes diverge gradually and acquire new functions (Ingram 1961). After the discovery that the IGS of rDNA is very similar among member genes of the same species but differs by ∼10% between different species in *Xenopus* (Brown *et al.* 1972), a new model of horizontal evolution was proposed and later termed “concerted evolution” (Zimmer *et al.* 1980). In the concerted model, all the repeats of a multigene family evolve as a unit in concert and mutations that occurred in a certain repeat will spread to all of the other repeats by unequal crossing over (Szostak and Wu 1980) or gene conversion (Hillis *et al.* 1991), resulting in greater sequence similarity within a species than between species (Dover 1982). The rDNA multigene families are subjected to concerted evolution in most eukaryotes (*e.g.*, Drouin and de Sá 1995; Koch *et al.* 2003; Nei and Rooney 2005; Ganley and Kobayashi 2007; Stage and Eickbush 2007). However, exceptions have been observed. Nonconcerted evolution of the ITS1-5.8S-ITS2 rDNA region has been demonstrated in *Mammillaria* (Harpke and Peterson 2006), *Pyrus* (Zheng *et al.* 2008), and a medicinal fungus *Ophiocordyceps sinensis* (Y. Li *et al.* 2013). Large numbers of polymorphisms were found in fungal ribosomal genes, indicating that they might not always evolve in a strictly concerted manner (Simon and Weiß 2008; Dakal *et al.* 2016). The coexistence of different classes of rRNA genes in the same genome was reported in various organisms such as plasmodia (Li *et al.* 1997; Mercereau-Puijalon *et al.* 2002; Rooney 2004), flat worms (Carranza *et al.* 1996), foraminifer (Pillet *et al.* 2012), oak trees (Muir *et al.* 2001), yeast (Dakal *et al.* 2016), and filamentous fungi (Rooney and Ward 2005). Pseudogenes are often found from those organisms in which rDNA evolves in a nonconcerted model (Zheng *et al.* 2008; Y. Li *et al.* 2013).

The proportion of ITS pseudogenic copies varies in different organisms. >97% of the ITS copies were pseudogenes in the cacti genus *Mammillaria*, whereas no pseudogenic ITS sequences were detected in the plant model species *Arabidopsis thaliana* (Harpke and Peterson 2007). Pseudogenic rDNA repeats are usually rare in fungi (Y. Li *et al.* 2013) and cannot be detected in a regular PCR; most fungal rRNA pseudogenes reported were cloned sequences (*e.g.*, Lindner and Banik 2011). Y. Li *et al.* (2013) developed specific primers to detect ITS pseudogenes in *O. sinensis*. However, this strategy has largely relied on the existing pseudogenes which could be used as references in primer design.

Though they have not been frequently discovered, rRNA pseudogenes may exist in more fungal species than previously expected. By benefitting from the increasing number of sequenced fungal genomes that cover broad fungal taxonomic groups, it is possible to discover potential rRNA pseudogenes using genome sequences. Therefore, the purpose of this study was to investigate the intragenomic polymorphisms of the 45S rDNA repeats in ascomycetes using genome data, and more importantly to identify rRNA (18S, 5.8S, and 28S) pseudogenes and evaluate their impact on phylogenetic analysis. The mutation types of identified rRNA pseudogenes were analyzed and the possible mechanism of pseudogene formation was also discussed.

## Materials and Methods

### Identification of rDNA repeats in WGS data

Sequences of ITS pseudogenes from *O*. *sinensis* (Y. Li *et al.* 2013) were used as queries to BLAST search the genome databases in GenBank to find fungal genomes that might contain rRNA pseudogenes. The searches were performed in May 2014 and discernable putative rRNA pseudogenes were found in nine genome assemblies of seven fungi, encompassing a diverse range of species in Pezizomycotina of Ascomycota, *i.e.*, two genome assemblies of *Neurospora crassa* (OR74A: PRJNA132 and PRJNA13841; Galagan *et al.* 2003), a widely used model organism; the recently published WGS data of *Cordyceps militaris* (CM01; Zheng *et al.* 2011), an insect pathogen; four genomes of three closely related *Epichloë* species, *i.e.*, *Epichloë amarillans* (E57), *E. brachyelytri* (E4804), and *E. typhina* (E8 and E5819); and genomes of two plant pathogens, *i.e.*, *Colletotrichum graminicola* (M1.001) and *Leptosphaeria maculans* (JN3; Rouxel *et al.* 2011). These seven species comprise both closely and distantly related fungal groups, suitable for a study of evaluating the impact of rRNA pseudogenes on phylogenetic analysis.

Genomic searches for all rDNA repeats were performed through repeatedly performing extensive local BLAST searches against each WGS data set of the nine genome assemblies, using rDNA sequences found in the preceding searches, until no new sequence was obtained. The sequences of the 18S-5.8S-28S rDNA unit were aligned with BioEdit version 7.0.9.0 (Hall 1999). Four sequences of 18S-5.8S-28S from *Epichloë* species were manually assembled based on the alignments and assigned as “contig EA” of *E*. *amarillans* (almost complete but lacking 48 bp at the end of 28S, assembled from two original contigs in the WGS data), “contig EB” of *E*. *brachyelytri* (complete, assembled from six original contigs), “contig E8-ET” of *E. typhina* E8 (complete, from two original contigs), and “contig E5891-ET” of *E. typhina* E5819 (almost complete but lacking 125 bp at the end of 28S, assembled from four original contigs) (Supplemental Material, Table S2 in File S1). Introns within 18S and 28S were identified and removed during the alignment.

Three rDNA sequences were retrieved from GenBank, including AB255603 and HM135162 of *C*. *militaris* (both incomplete) and FJ360521 of *N*. *crassa* (complete), and used as reference to functional gene sequences which were not found in the WGS data of those two species (Table S2 in File S1).

### GC content and sequence divergence levels

GC content was calculated with BioEdit for complete sequences of 18S, 5.8S, and 28S rRNA genes. Sequence divergence levels of each species were analyzed with the software MEGA5 (Tamura *et al.* 2011) using average *p*-distance. The distance analyses were performed with complete gene sequences of all the species except the functional 18S and 28S of *C. militaris* and 28S of *E*. *amarillans*, for which only partial sequences were available. Pairwise deletion was used for missing data treatment in the analyses of partial sequences.

### Mutation, repeat-induced point, and secondary structural analyses

Mutation analyses were performed with MEGA5. Sequences with the highest GC content of each species were used as references, either complete or incomplete according to the availability. Repeat-induced point (RIP) analyses were performed with the software RIPCAL (Hane and Oliver 2008) using sequences with the highest GC content as the consensus. The secondary structure model diagrams; *i.e.*, 18S rRNA of *N*. *crassa*, 5.8S and 28S rRNA of *Saccharomyces cerevisiae*; were obtained from the Comparative RNA Web Site and Project (http://www.rna.ccbb.utexas.edu/) and used as references. Secondary structures were drawn with the software Adobe Illustrator CS5.1 (Adobe Systems, San Francisco, CA).

### Identification and amplification of rRNA pseudogenes

Putative rRNA pseudogenes were identified by a combination of mutation and secondary structure analyses (Y. Li *et al.* 2013). To verify the pseudogenes identified from WGS data, experiments were designed to amplify them from living strains in the laboratory. Three fungal strains; *i.e.*, FGSC 10212 of *C. graminicola* (equivalent to M1.001 used for the genome sequencing; purchased from Fungal Genetic Stock Center [FGSC], University of Missouri, Kansas City, MO), FGSC 2489 of *N*. *crassa* (equivalent to OR74A, also from FGSC), and CGMCC 3.14242 of *C. militaris* (equivalent to CM01, purchased from China General Microbiological Culture Collection Center); were included in the test. Strains were incubated at 25° with potato dextrose agar in petri dishes for 15 d. Genomic DNA was then extracted from the cultured mycelia using a modified CTAB method (Yao *et al.* 1999). Specific primers for pseudogenes of 18S, 5.8S, and 28S (Table S4 in File S1) were designed based on sequence alignments of all rDNA paralogs. PCR amplifications were carried out in an ABI thermal cycler (Applied Biosystems) in a 100 μl reaction volume containing 50 μl 2× Taq PCR Master Mix (CWBIO Co., Beijing, China), 1 μl of each primer (10 μM), and 2 μl DNA template under the conditions of 5 min at 95°, 35 cycles at 95° for 1 min, 53° for 30 s, 72° for 1 min, and a final extension at 72° for 10 min. PCR products were purified using a Gel Extraction Kit (CWBIO Co.), following the manufacture’s instructions, and then cloned into pGEM-3Zf(+) vector (Promega, Madison, WI). The positive clones were sequenced using plasmid-specific primers M13F(−20) and M13R(−26) on Applied Biosystems (ABI) 3730 DNA sequencer by the Beijing Genomics Institute (Beijing, China).

### Phylogenetic analyses of three rRNA genes

The program MEGA5 was used to conduct the phylogenetic analyses of 18S, 5.8S, and 28S rDNA. Kimura two-parameter distances (Kimura 1980) were computed and used to generate neighbor-joining (NJ) trees. The statistical reliabilities of the internal branches were assessed for all trees by using 1000 bootstrap pseudoreplicates. Incomplete gene sequences were not used except contig EA (28S of *E*. *amarillans*), contig E5819-ET (28S of *E*. *typhina*), AB255603 (18S of *C*. *militaris*), and HM135162 (28S of *C*. *militaris*) because no complete functional genes were available from those species.

### Data availability

Sequences obtained in this study have been submitted to GenBank and the accession numbers are provided in the *Results*. File S2 shows the sequence alignments of ITS1, ITS2, and IGS regions of the seven species included in this study. File S3 shows the sequence alignments of 18S, 5.8S, and 28S rDNA regions which were used for phylogenetic analyses. Figure S1 contains the results of RIPCAL analyses of 18S, 5.8S, and 28S rDNA of five species that have not been shown in the main text. Table S1 in File S1 describes copy numbers of three rRNA genes in nine fungal genome assemblies. Table S2 in File S1 describes GC content of 18S, 5.8S, and 28S rDNA in the seven filamentous fungal species. Table S3 in File S1 describes C→T and G→A mutations in the 18S, 5.8S, and 28S rRNA pseudogene sequences. Table S4 in File S1 describes the specific primers for rRNA pseudogenes designed in this study.

## Results

### The 45S rDNA repeats extracted from nine genome assemblies of seven fungal species

A total of 211 sequences of the 45S rDNA repeat, including both complete (115) and partial (96) sequences, were retrieved from nine genome assemblies of seven fungal species (Table S1 in File S1). The number of rDNA repeats ranged from 2 to 91, with complete repeats ranging from 1 to 69, in the genome assemblies examined (Table S1 in File S1). There was only one complete and one partial repeat from the genome of *E. typhina* E8, the fewest among the nine assemblies. The most abundant rDNA repeats were found in the genome of *L. maculans* (69 complete and 22 partial repeats) and in the reference genome of *N. crassa* (PRJNA132; 16 complete and 53 partial repeats) (Table S1 in File S1).

### GC content and sequence divergences

Comparison of the three rDNA subunits revealed that the GC content of different rDNA paralogs varied greatly in the species examined (Table S2 in File S1). For example, GC content in *N*. *crassa* (PRJNA132 and PRJNA13841) varied from 22.61 to 48.39%, 12.26–45.81%, and 25.36–51.26% in the sequences of 18S, 5.8S, and 28S genes, respectively. The highest GC content of complete sequences of 18S, 5.8S, and 28S genes were 48.47% (*E*. *brachyelytri*), 48.39% (*C*. *militaris*), and 52.55% (*C. graminicola*), respectively; while the lowest were 22.61% (*N. crassa*, PRJNA132), 12.26% (*N. crassa*, PRJNA132, and *E*. *brachyelytri*), and 25.36% (*N. crassa*, PRJNA132). The sequences of an individual gene from each species could be divided into two groups based on unoverlapped GC content, *i.e.*, 46.45‒52.55% and 12.26‒44.43% (Table S2 in File S1).

Sequence divergence analyses showed that the average *p*-distance values of rDNA paralogs ranged from 0.057 to 0.143 (average 0.109) for 18S, 0.062 to 0.181 (average 0.137) for 5.8S, and 0.053 to 0.161 (average 0.117) for 28S in all the seven species; indicating a high divergence level (Table 1).

**Table 1:** Sequence divergences and mutation rates of the three rRNA coding genes

Species	Average *p*-Distance			G→A Mutation Rate			C→T Mutation Rate		
	18S	5.8S	28S	18S	5.8S	28S	18S	5.8S	28S
*C. militaris*	0.14	0.18	0.16	0.10	0.12	0.12	0.08	0.12	0.09
*E. amarillans*	0.08	0.10	0.09	0.08	0.11	0.09	0.06	0.08	0.07
*E. brachyelytri*	0.11	0.15	0.12	0.10	0.14	0.11	0.08	0.13	0.09
*E. typhina*	0.12	0.16	0.12	0.09	0.14	0.12	0.08	0.13	0.10
*C. graminicola*	0.11	0.15	0.12	0.09	0.12	0.09	0.08	0.13	0.09
*L. maculans*	0.06	0.06	0.05	0.07	0.09	0.08	0.06	0.07	0.06
*N. crassa*	0.14	0.16	0.16	0.10	0.14	0.12	0.12	0.14	0.12
Average	0.11	0.14	0.12	0.09	0.12	0.10	0.08	0.11	0.09

### Mutation and secondary structure analyses

**Figure 1:**
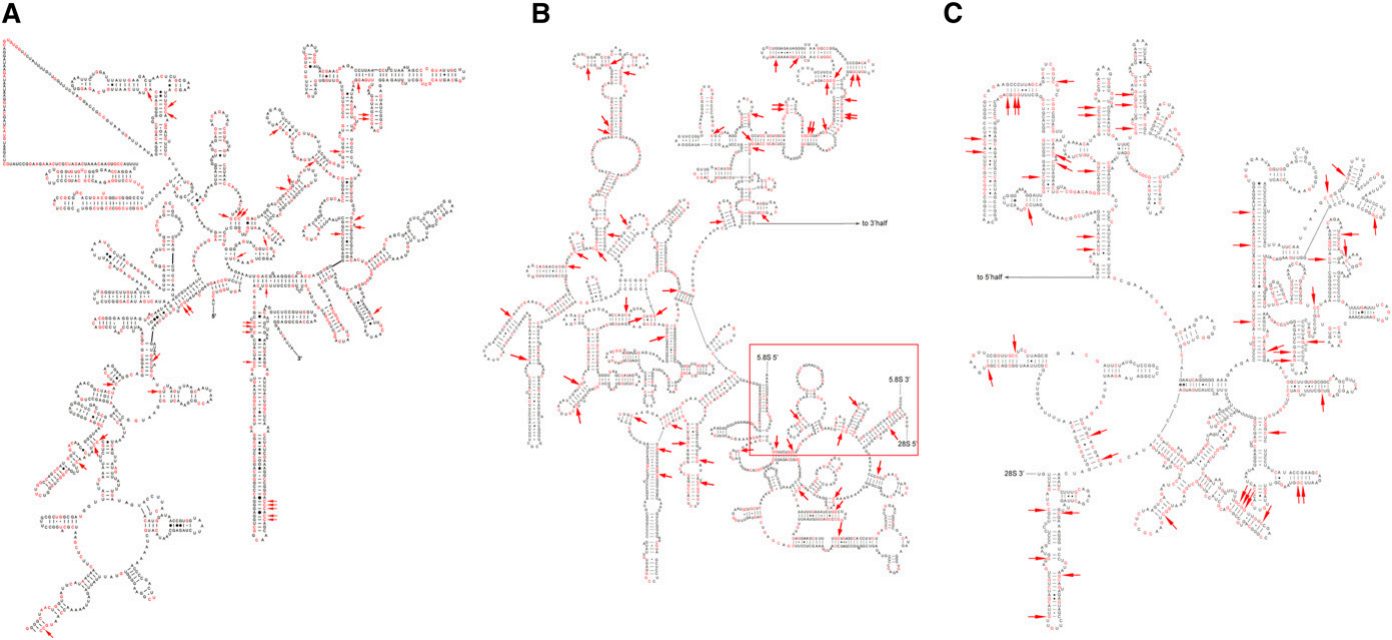
(A) Secondary structures of 18S rRNA gene, (B) 5′ half of 28S rRNA gene with 5.8S rRNA binding on it (marked in red frame), and (C) 3′ half of 28S rRNA gene. Structural diagrams were drawn with a GenBank sequence of *N. crassa* (accession number FJ360521). The 5.8S rRNA is shown in red frame. Observed mutated base pairs in pseudogenes were marked in red. Mutations which would probably destroy the secondary structures were marked with red ↓.

Nucleotide mutation types were analyzed for the rDNA paralogs with a low GC content; it showed that G→A and C→T transitions were the two main mutation types (Table S2 in File S1). Other mutation types; *i.e.*, indels, A→G and T→C transitions, and all the transversions (A↔T, A↔C, T↔G, and G↔C); occurred at a very low frequency (<0.1%, data not shown), equivalent to the error rate of amplification and sequencing (Shendure and Ji 2008). The secondary structures of 18S, 5.8S, and 28S rRNA gene of *N*. *crassa* predicted using a GenBank sequence (accession number FJ360521) were shown in Figure 1. Structures of sequences with no transition mutations among seven species were highly similar and accordant with the eukaryotic structure models (Ben-Shem *et al.* 2010). It was showed that G→A and C→T transitions were not confined to unpaired regions but were also found in the conserved structural regions, *i.e.*, loops in helices, which could probably destroy the secondary structures (Figure 1).

### Identification and amplification of rRNA pseudogenes

As in the case of *O. sinensis* (Y. Li *et al.* 2013), ITS sequences with a lower GC content caused by C→T and G→A mutations were not expressed. Further, C→T and G→A transition mutations could probably destroy the secondary structures. Sequences with a lower GC content showing uniform C→T and G→A mutations in the WGS data were thus determined as pseudogenes in this study. The number of pseudogenes ranged from 2 in *C. militaris* to 89 in *L*. *maculans* (Table S2 in File S1). These pseudogenic sequences were verified through a procedure of PCR amplification using specifically designed primers, followed by cloning of amplicons and sequencing. The amplifications were successful in *C. graminicola* and *C. militaris*, recovering parts of pseudogenic sequences of 18S, 5.8S, and 28S identified from their WGS data. Amplifications of 18S, 5.8S, and 28S pseudogenes were unsuccessful for *N. crassa* FGSC 2489 (equivalent to *N. crassa* OR74A) even though four combinations of two different primers from both ends were used (Table S4 in File S1). The pseudogenic sequences obtained have been submitted to GenBank under the accession numbers KP215645–KP215656.

### RIP analyses

**Figure 2:**
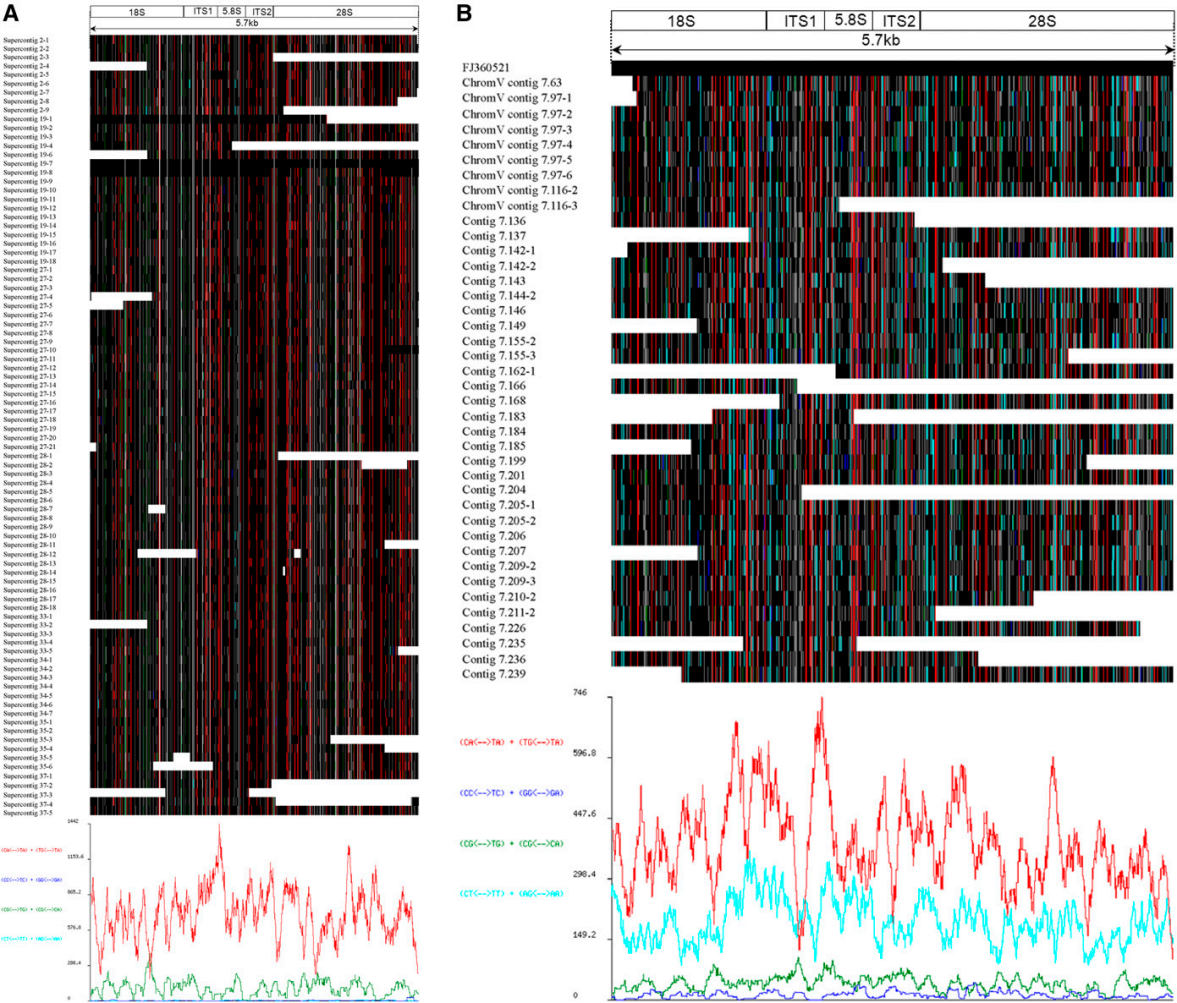
RIP mutation in rDNA units of (A) *L. maculans* and (B) *N. crassa* shown as RIPCAL output. Functional rDNA sequences, i.e., supercontig 19-7 of *L*. *maculans* and FJ360521 of *N*. *crassa* were defined as consensus. Black, invariant nucleotide; white, gap; red, CpA ↔ TpA or TpG ↔ TpA mutations; dark blue, CpC ↔ TpC or GpG ↔ GpA mutations; green, CpG ↔ TpG or CpG ↔ CpA mutations; pale blue, CpT ↔ TpT or ApG ↔ ApA mutations.

The results of analyses using RIPCAL showed that RIP mutations (represented by high rate of G→A and C→T transitions) distributed evenly in most of the rDNA pseudogenic sequences (Figure 2 and Figure S1), but varied in some cases, *e.g.*, present at parts of 28S in supercontig 19-8 or absent from a part of 28S in supercontig 27-10 of *L*. *maculans* (Figure 2A). The average rates of RIP mutations for three rDNA subunits were from 17.25% (18S) to 23.60% (5.8S) for all the species examined, with the lowest rate in *L. maculans* (12.78‒15.56%) and the highest in *N. crassa* (22.80‒28.00%) (Table S3 in File S1). Interestingly, the rates were generally higher in the six species of Sordariomycetes, 13.89‒28.00%, than in *L. maculans* (Dothideomycetes), and were higher in 5.8S (total average 23.60%) than in 18S and 28S (total average 17.25% and 19.15%, respectively) in all the species examined (Table 1 and Table S3 in File S1). The number of RIP mutations in individual rDNA paralogs, including both G→A and C→T mutations, was generally close to each other within species, but exceptional low numbers (often less than or around a half of the normal numbers) were also found, *e.g.*, the three rDNA subunits in contig 00227 of *E*. *brachyelytri*, 18S in contig 00515 of *E*. *typhina* E8, 5.8S in supercontig 2-5 of *L. maculans*, and 28S in contig 00535-1 of *E. typhina* E5819 (Table S3 in File S1). Further, mutation rates of G→A were usually higher than that of C→T in the three rDNA subunits of the species analyzed, but slightly lower in 18S and 28S of *N. crassa* and in 5.8S of *C. graminicola* (Table 1 and Table S3 in File S1).

In addition to the three rDNA subunits, other components of the 45S rDNA repeats, including ITS1, ITS2, and IGS, have also been affected by RIP; showing a similar mutation pattern of mainly C→T and G→A transitions (Table S3 in File S1).

The RIPCAL outputs (Figure 2 and Figure S1) indicated that CpA and TpG dinucleotides were mutated more frequently to TpA rather than CpC, CpG, CpT, GpG, CpG, and ApG dinucleotides (Figure 2 and Figure S1). CpT and ApG dinucleotides were exceptionally mutated to TpT and ApA with a comparatively higher rate in *N. crassa* (Figure 2B) than in the other species (Figure 2A and Figure S1), indicating different mutation bias in different species.

### Phylogenetic analyses

**Figure 3:**
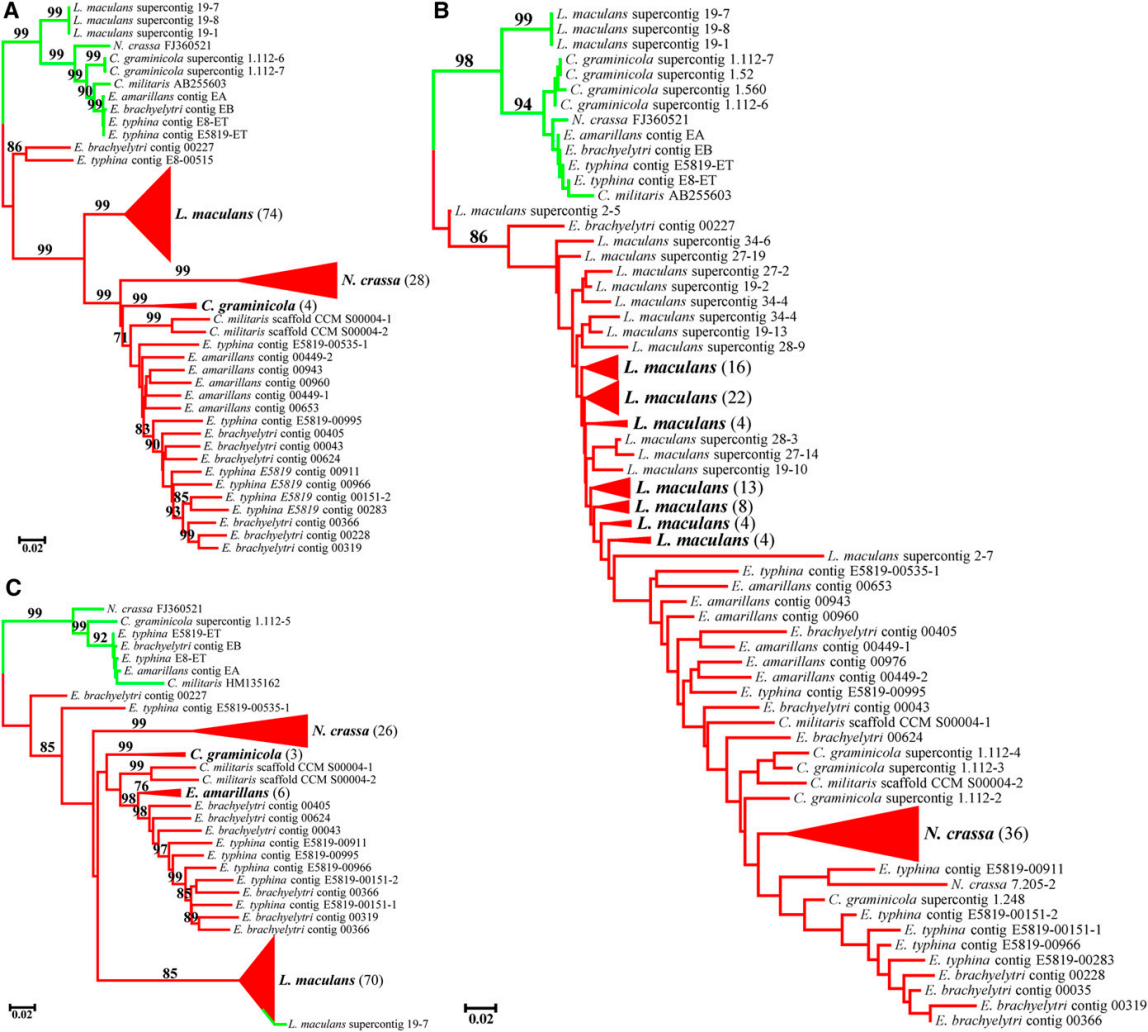
Cladogram of the (A) 18S, (B) 5.8S, and (C) 28S rDNA paralogs from seven fungal species examined in this study using NJ analyses. Blue and red branches indicate functional genes and pseudogenes, respectively. Numbers in brackets are rDNA sequences included in each compressed clade. Bootstrap support values that were <70% were not shown.

The data sets for phylogenetic analyses consisted of 138, 161, and 128 sequences for 18S, 5.8S, and 28S genes, respectively (see the alignments, File S3). The distance-based tree construction method, NJ, was used to reconstruct cladograms. The sequences were mostly clustered into two major clades representing two gene types: functional and pseudogenic. Sequences of the three rRNA genes were not grouped by taxonomic species but were intermixed (Figure 3). Functional gene and pseudogenic sequences from the same species clustered into two separate clades in all the analyses, except for the 28S gene in which the functional gene of *L. maculans* grouped in the pseudogenic clade (Figure 3C).

For the 18S gene, 11 functional genes and 127 pseudogenes were well separated into two distinct clades (Figure 3A). Functional gene sequences from a species were found to be identical among different copies within a genome (*i.e.*, *C. militaris* and *C. graminicola*), but might vary in different genomes of one species (*i.e.*, *E. typhina* E8 and E5819), and even intermixed with closely related species (*i.e.*, three *Epichloë* species: *E. amarillans*, *E. brachyelytri*, and *E. typhina*); while pseudogenes were much more divergent within or between species (Figure 3A). Pseudogene sequences formed well-supported clades for distantly related species (*i.e.*, *C. militaris*, *C. graminicola*, *L. maculans*, and *N. crassa*), whereas they displayed an interspecies clustering pattern for closely related species (*i.e.*, *E. amarillans*, *E. brachyelytri*, and *E. typhina*). In particular, 18S sequences in contig 00227 of *E*. *brachyelytri* and contig 00515 of *E*. *typhina* E8 were separated from all the other sequences of the same species, forming a basal branch of the pseudogene clade (Figure 3A).

A total of 13 functional genes and 148 pseudogene sequences were included in the analyses of the 5.8S gene (Figure 3B). Similarly, two distinct clades of functional genes and pseudogene sequences were formed that were irrelevant to the species origin of the sequences. For the functional genes, two well-supported clades were formed, where the most distantly related species, *L. maculans* (Dothideomycetes), were separated from the others (Sordariomycetes). Among Sordariomycetes, there were some groupings of sequences, *e.g.*, the sequences of *C*. *graminicola* clustered together and separated from the remaining species, although there is no support for the clusters. Within the 5.8S pseudogene clade, the sequences were intermixed without clear grouping by species; but all of the sequences from Sordariomycetes clustered together in a terminal clade, except for the sequence in contig 00227 of *E*. *brachyelytri*. Similarly, the sequences from *L. maculans*, except that in supercontig 2-5 of *L. maculans*, formed a basal group paraphyletic to the Sordariomycetes clade (Figure 3B).

In the 28S gene analysis, eight functional genes and 122 pseudogene sequences were included. Functional gene sequences from six of the seven species grouped in a very strongly supported clade, except that from *L. maculans* which was embedded within the pseudogene clade of the same species. In the pseudogene clade, sequences from all the species but *E. brachyelytri* and *E. typhina* were grouped by species with reasonable to very strong supports (Figure 3C). Most sequences from *E. brachyelytri* and *E. typhina* formed a mixed clade, but two of them, in contig 00227 of *E*. *brachyelytri* and contig 00535-1 of *E. typhina* E5819, respectively, were placed in the basal branches of the pseudogene clade (Figure 3C).

## Discussion

### rRNA pseudogenes identification and validation

Previous studies indicated that rDNA underwent concerted evolution in fungi (*e.g.*, Ganley and Kobayashi 2007). However, the 5S rRNA multigene family was found to evolve in a birth-and-death model in filamentous fungi with 18–83% of the rDNA sequences identified as pseudogenes (Rooney and Ward 2005). ITS pseudogenes were also reported from the medicinal fungus *O. sinensis* (Y. Li *et al.* 2013). Secondary structure stability and patterns of nucleotide substitutions in the coding regions are the two criteria for distinguishing putative pseudogenes from presumed functional sequences (Razafimandimbison *et al.* 2004). The truncated 5S gene sequences were identified to be pseudogenes because of the lack of an intact coding sequence, which effectively destroys the secondary structure of the 5S rRNA (Rooney and Ward 2005). Y. Li *et al.* (2013) analyzed the secondary structure and minimum free energy of the 5.8S rRNAs in *O*. *sinensis* and found that ITS sequences, having a lowered GC content caused by a number of G:C to A:T transition mutations, were pseudogenes which were not expressed in the reverse transcriptional test. Analyses of WGS data of the seven filamentous ascomycetes included in this study showed a high level of sequence variation within rDNA arrays (Table 1). G:C to A:T transition mutations were found in a large portion of rDNA paralogs, resulting in an obviously lowered GC content. Those sequences were identified as putative pseudogenes. Pseudogenes of the rRNA multigene families may exist in a wider range of biological fungal groups than previously suspected.

Intragenomic variations, including pseudogenes observed in high-throughput pyrosequencing or detected occasionally, were suspected to be caused by amplification and sequencing errors (Lücking *et al.* 2014a, b) or by the impurity of sequence chromatograms (Muggia *et al.* 2014). A cloning and sequencing strategy was thus adapted in the present study to confirm the presence of rRNA pseudogenes in the fungal genomes observed. On the basis of the rRNA pseudogenes in WGS, specific primers were designed to detect them in living strains. Strains of *C. graminicola*, *C. militaris*, and *N. crassa* used for genome sequencing were obtained. The amplification of the rRNA pseudogenes using the specifically designed primers was successful in the first two species but failed in *N. crassa*. Sequencing of the cloned amplicons proved the authenticity of rRNA pseudogenes identified from the WGS data. The failure of amplification in *N. crassa* could possibly be caused by numerous point mutations of AT bias in those targeted pseudogenic sequences, resulting in an extremely high AT content in the specifically designed primers (Table S4 in File S1) which made the PCR amplification inefficient. It is worth mentioning that the aim of the amplification by specific primers was to verify the pseudogenes observed from the WGS data. It may be possible to find all pseudogenes in the rDNA paralogs by pyrosequencing.

### Effects of rRNA pseudogenes on fungal phylogeny

Various regions of rDNA have been used as molecular markers in phylogenetic analyses and the ITS has been selected as the standard bar code in general for fungal DNA bar coding (Seifert 2009; Schoch *et al.* 2012). However, usually only functional sequences were included in such studies. The utility of the rRNA pseudogenes in the phylogenetic analyses is often debated. Previous studies showed that rRNA pseudogenes provided useful information for phylogenetic analyses of closely related species and could be used as better outgroups than sister species (Ochieng *et al.* 2007). They are even more useful for groups without closely related extant taxa (Buckler and Holtsford 1996a, 1996b; Buckler *et al.* 1997). However, on the other hand, rRNA pseudogenes could accumulate mutations and cluster randomly across phylogenetic trees, confounding attempts to infer correct phylogenetic relationships (*e.g.*, Kita and Ito 2000; Mayol and Rosselló 2001; Xiao *et al.* 2010). For example, 5S rDNA paralogs in fungi showed an interspecies clustering in closely related species (Rooney and Ward 2005).

As in the seven filamentous ascomycetes studied here, the interspecies clustering pattern in NJ analyses of the three rRNA coding genes would cause problems in species identification and phylogenetic reconstruction. Sequences of functional genes and pseudogenes generally formed separate clades in the NJ analyses, with the only exception being 28S in supercontig 19-7 of *L. maculans*, which was imbedded with its pseudogenic counterparts (Figure 3). The possible reason for this could be the comparably distant relationship between *L. maculans* and the other species included in the analyses; *L. maculans* belongs to the class Dothideomycetes while the others are members of Sordariomycetes. The intrinsic difference of 28S functional gene sequences between the species included was greater than that between functional genes and pseudogenes within the genome of *L. maculans*. Several pseudogenic sequences (contig 00227 of *E. brachyelytri*, contig 00515 of *E. typhina* E8, supercontig 2-5 of *L. maculans*, and contig 00535-1 of *E. typhina* E5819) were also not falling into the main pseudogenic clade but in separate basal branches close to the functional gene clade (Figure 3). This could possibly be caused by the fact that limited RIP mutations occurred in those sequences (Table S3 in File S1).

Based on the results of this study, it is necessary to clarify the possible pseudogenes for accurate phylogenetic analyses and species identification. When pseudogenes are present, phylogenetic estimation methods, like maximum-parsimony (MP) and Bayesian analyses, may not be able to establish the correct phylogenetic relationship between taxa. The default substitution rates of the three evolutionary models, *i.e.*, F81, HKY, and GTR, implemented in Bayesian analyses are not consistent with the RIP mutation frequencies. It was reported that >100 G:C to A:T transition mutations could accumulate in a 9-kb sequence after several rounds of sexual reproduction (Graïa *et al.* 2001). Moreover, as presented in the results of this paper, the point mutations and mutation rates of RIP varies irregularly among different copies of pseudogenes and therefore it is difficult to fix the substitution rates for the evolutionary models. Similarly, the nucleotide mutation assumption in MP analyses is also not in concordance with the RIP process.

### RIP mutation of rRNA pseudogenes

Three types of pseudogenes, including proceeded (retrotransposed), nonproceeded (duplicated), and unitary, have been reported (W. Li *et al.* 2013). For the nonproceeded pseudogenes, a probable mechanism termed RIP has been experimentally demonstrated in various fungal species (Graïa *et al.* 2001; Ikeda *et al.* 2002; Galagan and Selker 2004; Coleman *et al.* 2009). RIP is a mechanism that can inactivate genes through introducing numerous G:C to A:T transition mutations during sexual reproduction (*i.e.*, meiosis; Selker *et al.* 1987). It was first discovered in 5S rDNA in a fungal species *N*. *crassa* (Selker *et al.* 1987) and was considered to be unique to fungi (Galagan *et al.* 2003). RIP mutates duplicated sequences, either linked or unlinked, longer than ∼400 bp (or ∼1 kb in the case of unlinked duplications) with a similarity greater than ∼80% (Cambareri *et al.* 1991; Watters *et al.* 1999). RIPCAL analyses of the rRNA pseudogenes in nine genome assemblies showed that G→A and C→T transitions distributed randomly in the whole rDNA pseudogenic repeats. All seven filamentous ascomycetes studied are affected by RIP, including four reported previously; *i.e.*, *C. militaris* (Zheng *et al.* 2011), *C. graminicola* (Clutterbuck 2011), *L. maculans* (Rouxel *et al.* 2011), and *N. crassa* (Galagan *et al.* 2003); and three reported here, *i.e.*, *E*. *amarillans*, *E*. *brachyelytri*, and *E*. *typhina*.

RIPs have been found in various fungi in transposable elements (*e.g*., Neuveglise *et al.* 1996; Hood *et al.* 2005; DiGuistini *et al.* 2011), but are not often reported in the rDNA repeats. Theoretically, the 45S rDNA repeats of filamentous ascomycetes are perfect candidates for RIP as they are congruent with the required characteristics (>400 bp with a sequence identity of >80% [Cambareri *et al.* 1991; Watters *et al.* 1999]). It has been suggested that the tandem rDNA repeats in the nucleolar organizer regions (NORs) were protected from RIP (Selker 1990) and this was confirmed in the *N*. *crassa* genome where RIP was found only in several copies located outside the NORs (Galagan *et al.* 2003). However an exceptional case was reported for *L. maculans* with extensively affected rDNA repeats, but the location of each rDNA repeats was not specified (Rouxel *et al.* 2011). In *Stagonospora nodorum*, a repeat at the array terminus also showed evidence of RIP at similar levels to those of non-rDNA-array repeats (Hane and Oliver 2008). It is important to locate both functional and RIP-mutated rDNA repeats to investigate the relationships between RIP and the location of rDNA on chromosomes. However, little information about the chromosomal location of different rDNA paralogs has been acquired for the species included in this study. There is not enough evidence at this stage to test whether all rDNA repeats can be equally affected, or whether only those outside the NORs are affected by RIP in those species. Moreover, it is unclear whether the partial effects of some rDNA repeats, *e.g.*, supercontigs 19-8 and 27-10 of *L. maculans* (Figure 2A), are true or they are simply errors that occurred in genome assembly. Further studies are needed to test these possibilities.

### rDNA copies and genome assembly

Among the nine genome assemblies investigated, the greatest number of rDNA copies was found in *L. maculans* JN3 (91, Table S1 in File S1), of which 89 were pseudogenes. There were 69 rDNA copies in the PRJNA132 assembly of *N. crassa* OR74A, of which all were pseudogenes but one was an incomplete functional gene. For the other assemblies, much fewer copies were found, from 2 in *E. typhina* E8 to 11 in *C. graminicola* M1.001 (Table S1 in File S1). Genome assembly may underestimate the number of repetitive DNA sequences. Functional rDNA sequences are often assembled into one or fewer repeats because of the high similarity among different copies and the short-read length of the current sequencing technology (James *et al.* 2009). Pseudogenes resulting from RIP are comparatively easier to be maintained as they possess a large number of variations which prevent different copies being assembled into one. Consequently, pseudogene copies seem to be more prevalent than functional ones in the WGS data. However, the number of rDNA repeats obtained from WGS data may not reflect the real copies in a genome; the proportion of rRNA pseudogenes and functional rDNA repeats cannot be estimated by the genome sequences currently available. Short-read length assemblies with conflated rDNA repeat sequences would require special bioinformatic methods to reliably predict the functional version. A possible solution could be to select the version with the highest GC content or with the highest read coverage from a stringent read alignment (*i.e.*, not allowing for SNPs).

## Supplementary Material

Supplemental material is available online at www.g3journal.org/lookup/suppl/doi:10.1534/g3.117.044016/-/DC1.
