# Supplementary material for: rRNA Pseudogenes in Filamentous Ascomycetes as Revealed by Genome Data
Source: G3 (Bethesda). 2017 Jun 21;7(8):2695–703. doi: 10.1534/g3.117.044016 (PMC5555474; doi:10.1534/g3.117.044016)
Supplement: Supplementary file 1 [file 2695FigureS1.doc]

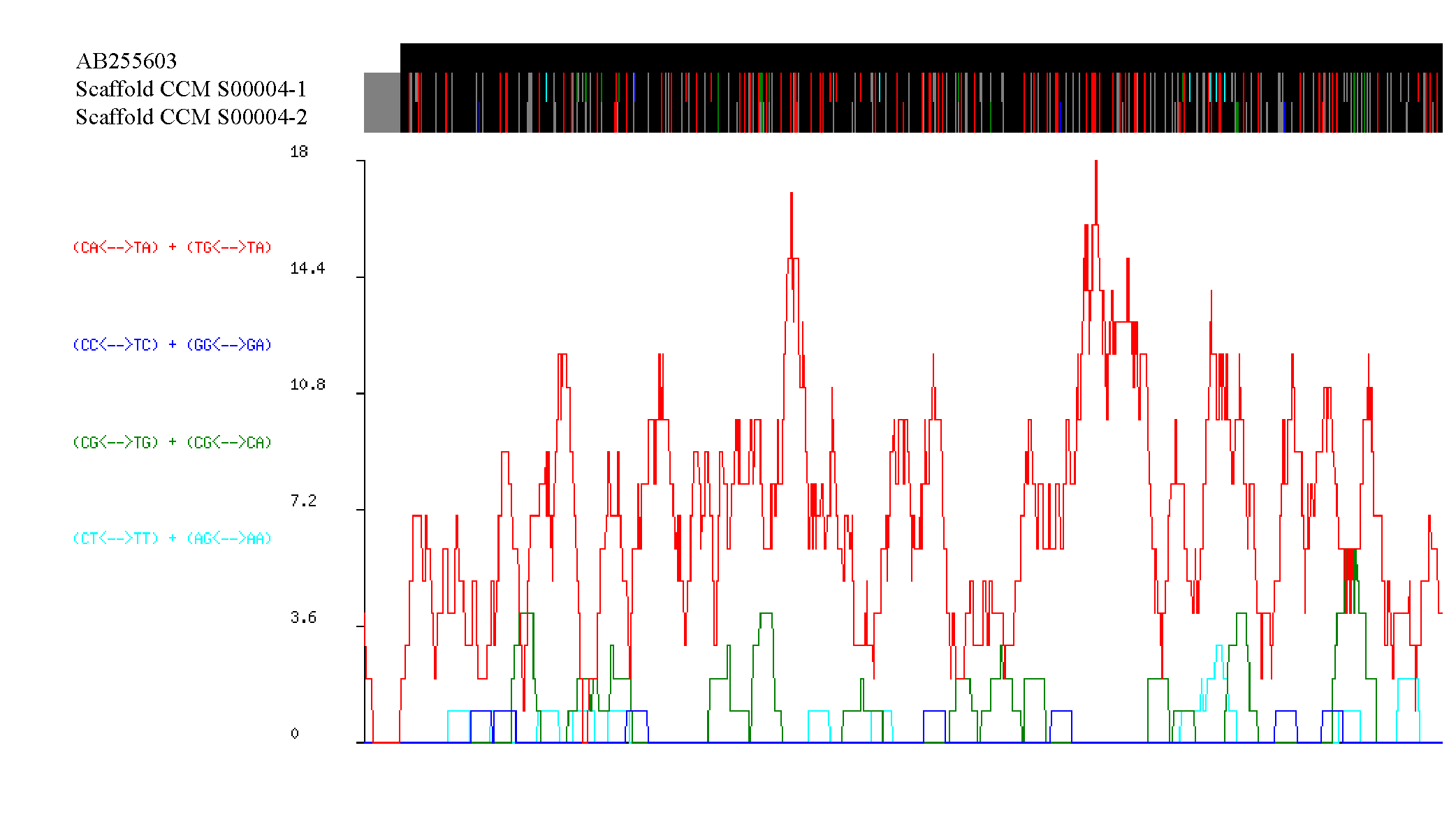


**Fig. A.1-1**. RIP in 18S of *Cordyceps militaris*.


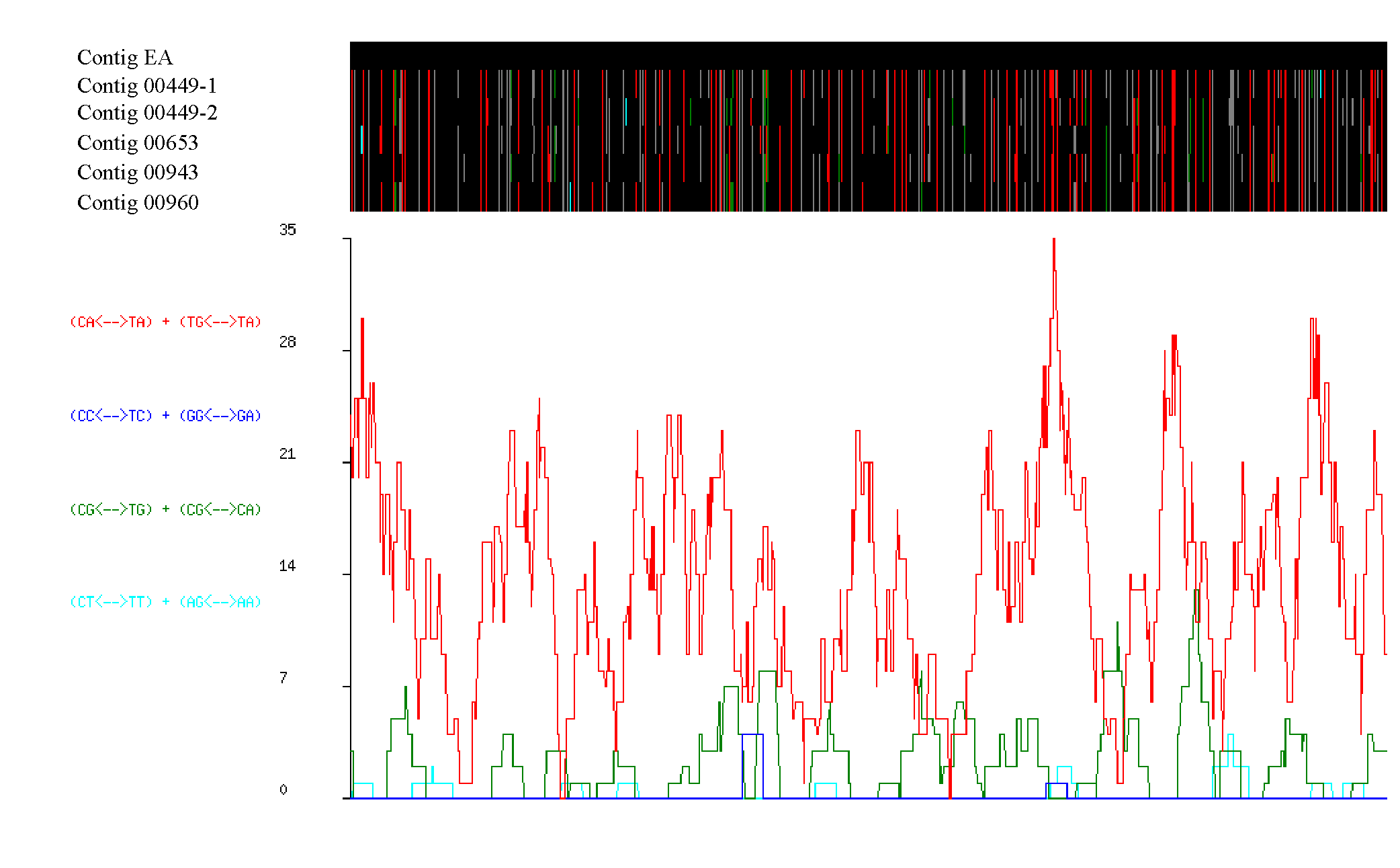


**Fig. A.1-2**. RIP in 18S of *Epichloë amarillans*.


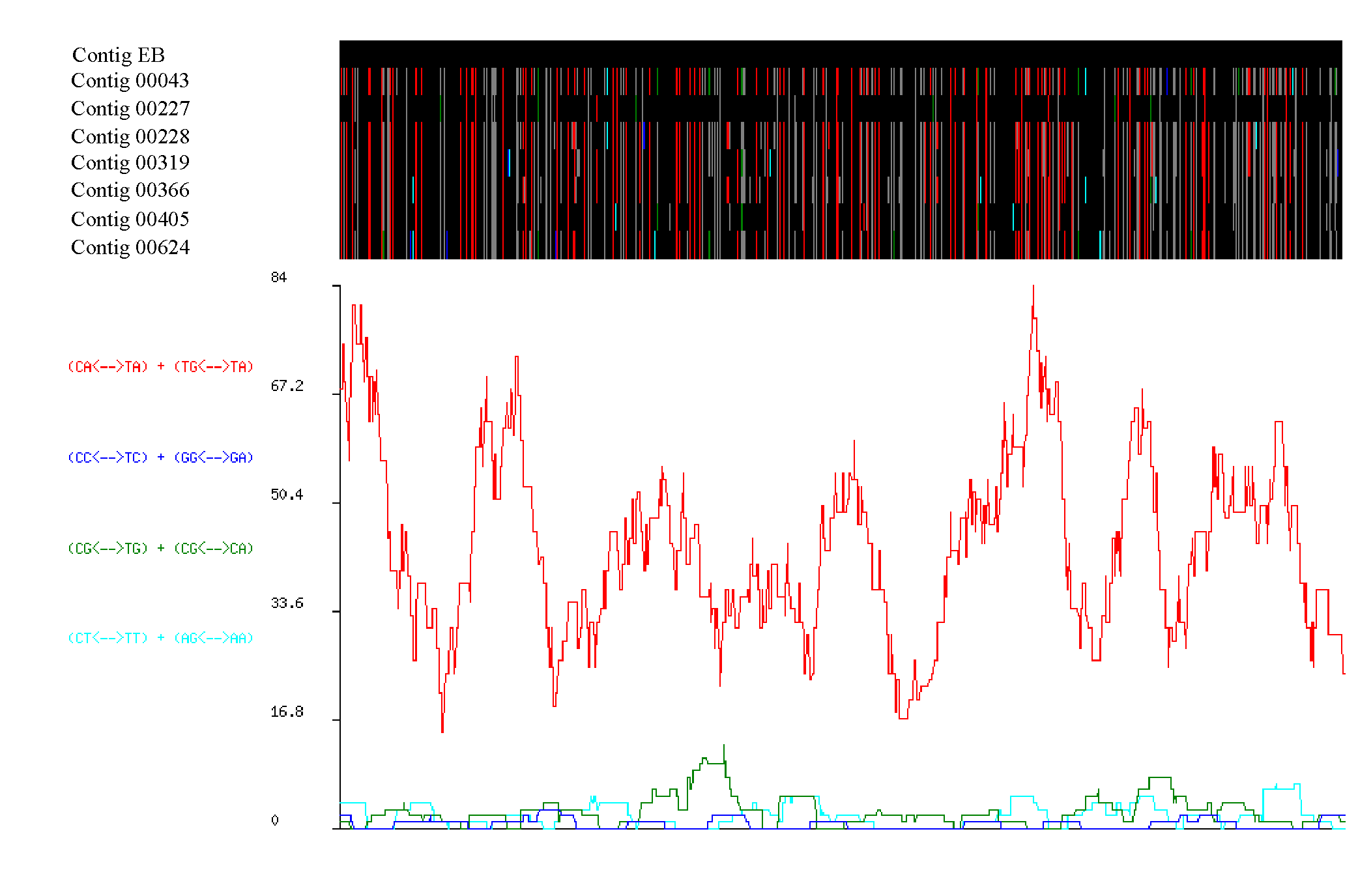


**Fig. A.1-3**. RIP in 18S of *Epichloë brachyelytri*.


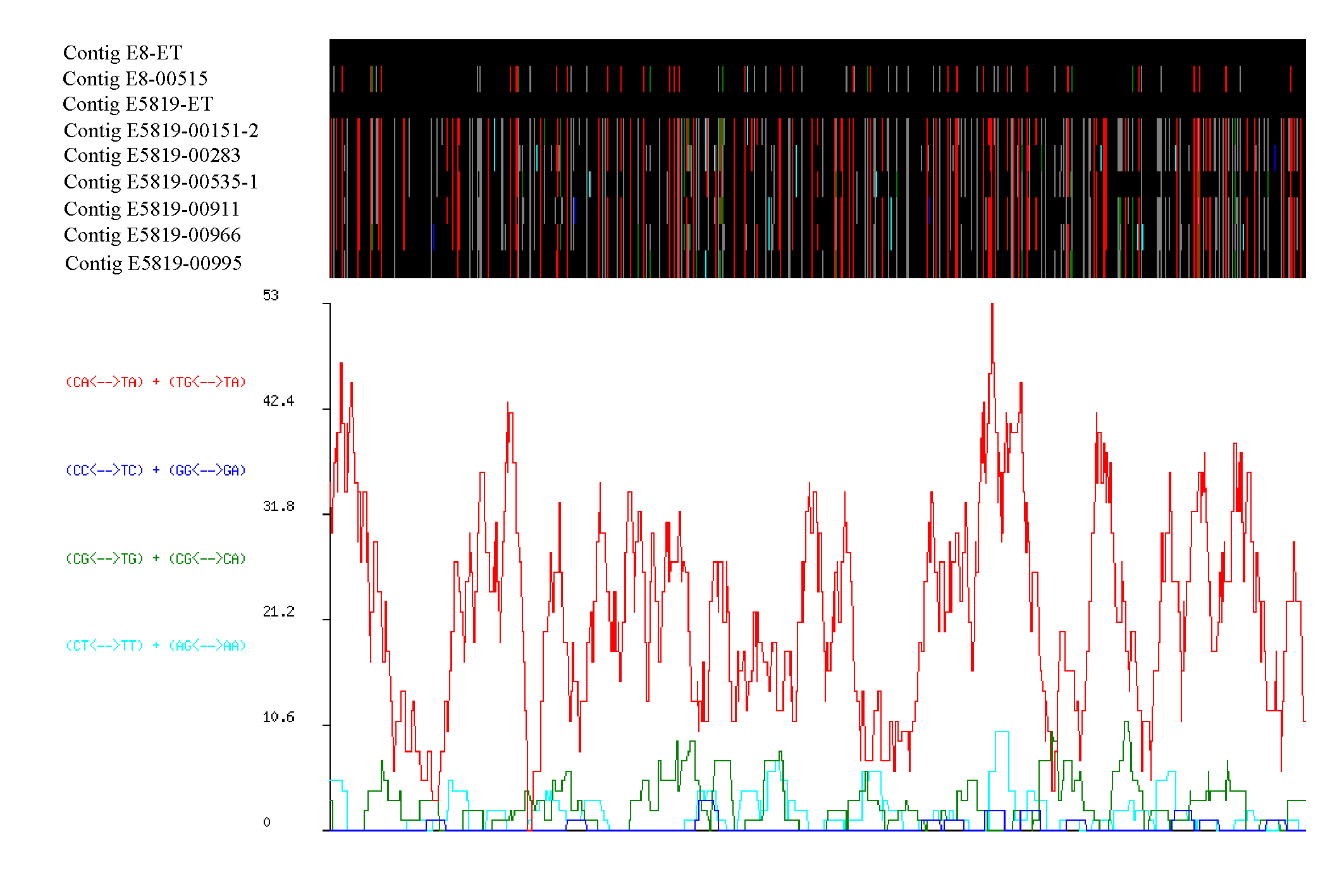


**Fig. A.1-4**. RIP in 18S of *Epichloë typhina*.


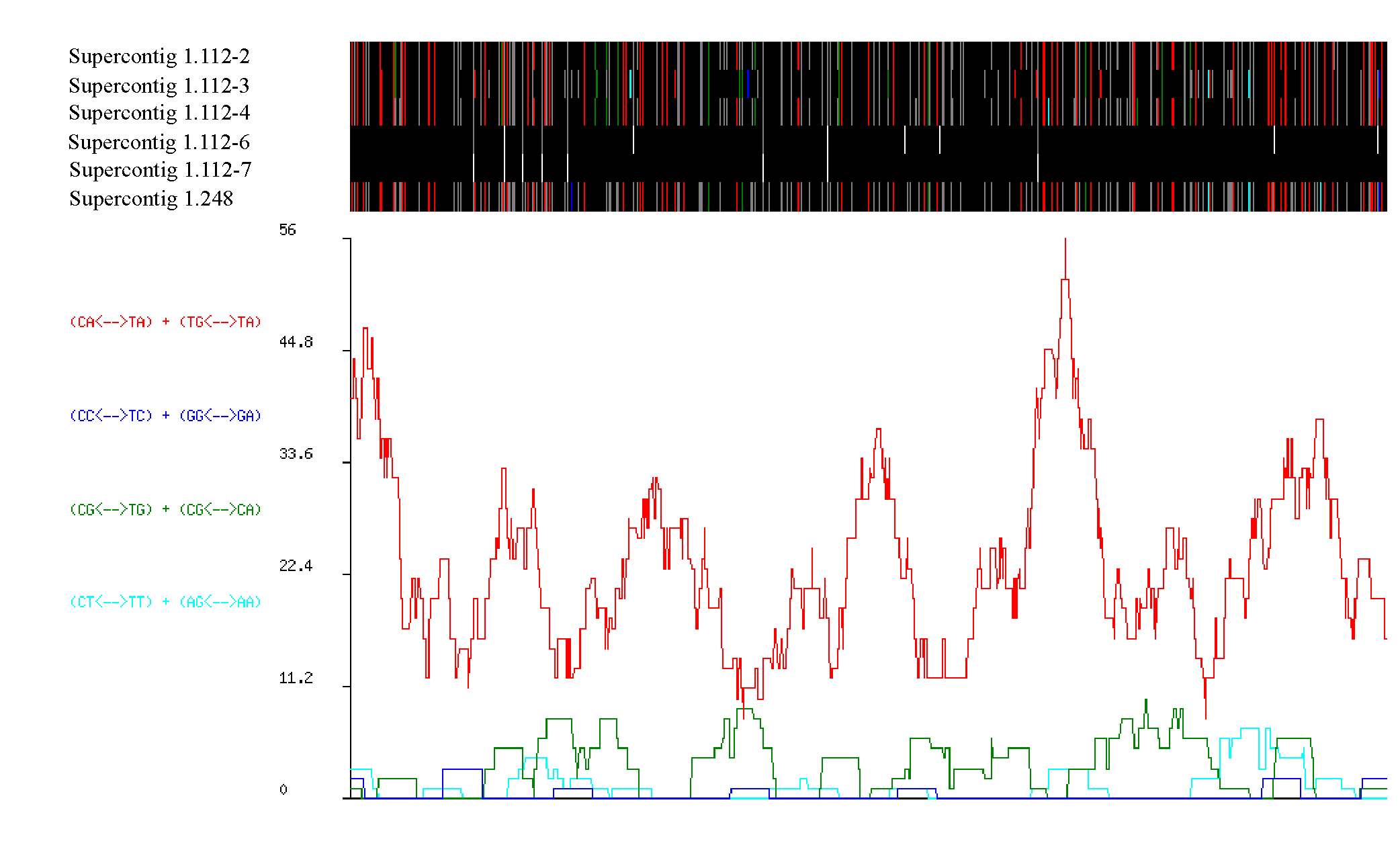


**Fig. A.1-5**. RIP in 18S of *Colletotrichum graminicola*.


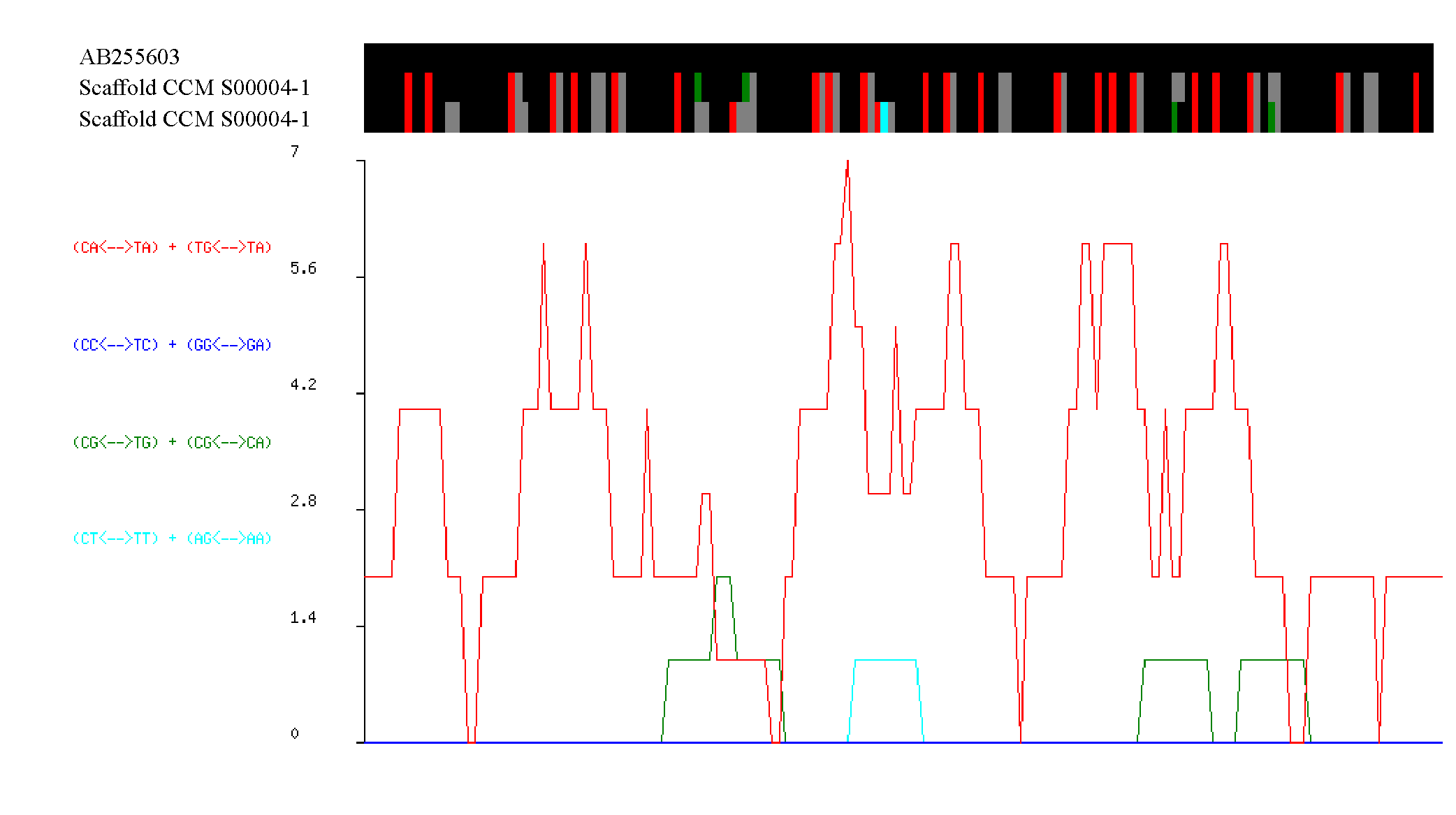


**Fig. A.1-6**. RIP in 5.8S of *Cordyceps militaris*.


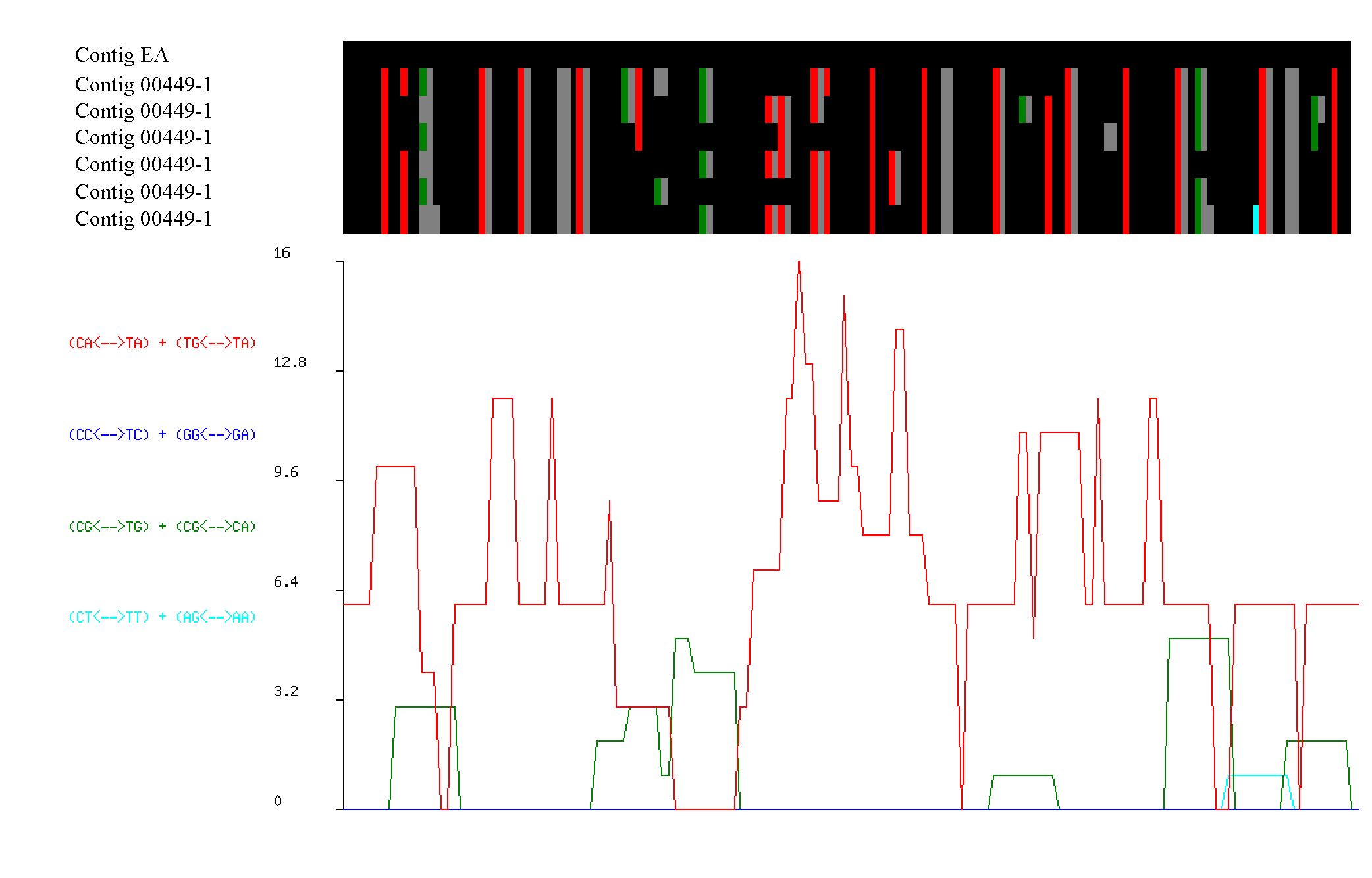


**Fig. A.1-7**. RIP in 5.8S of *Epichloë amarillans*.


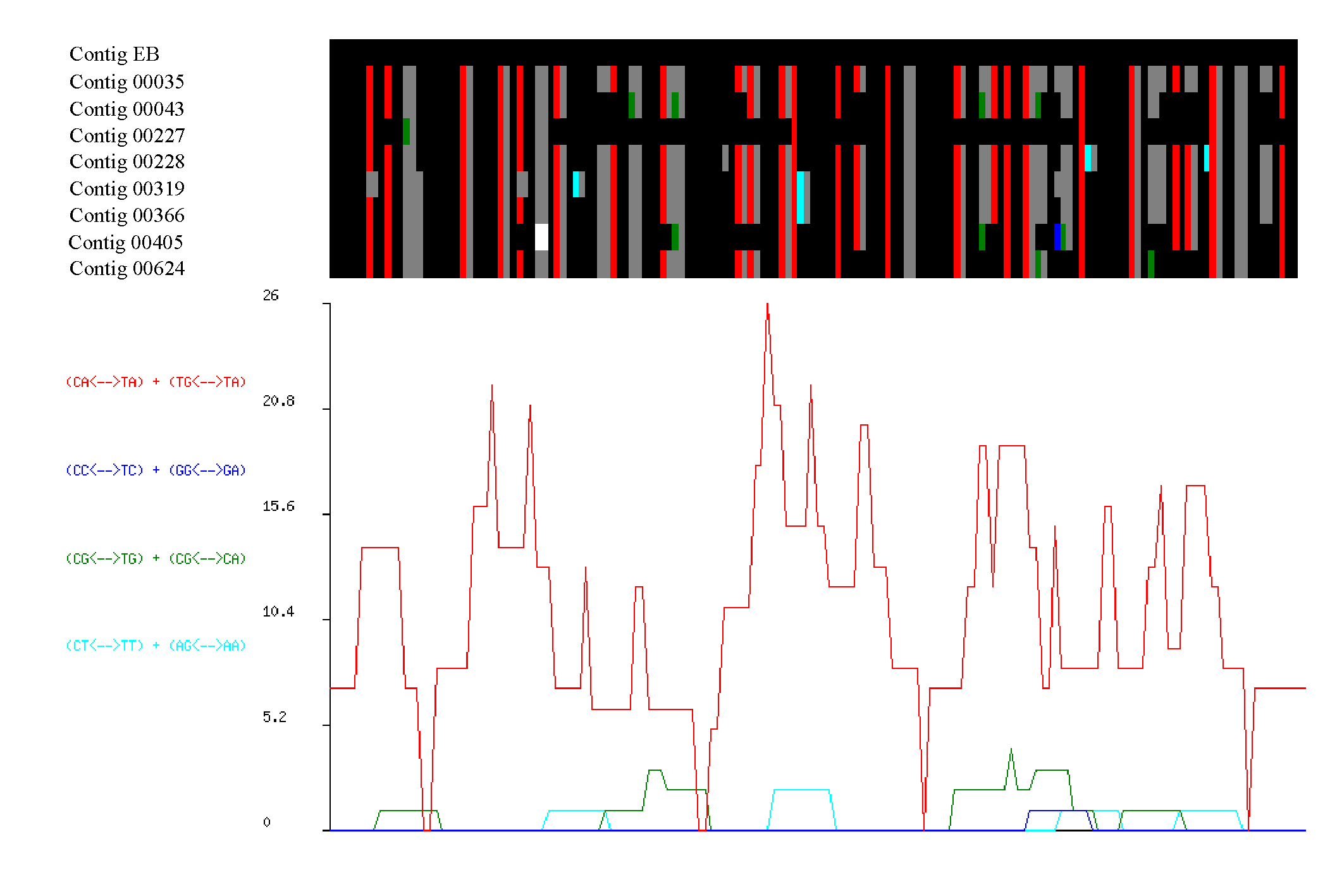


**Fig. A.1-8**. RIP in 5.8S of *Epichloë brachyelytri*.


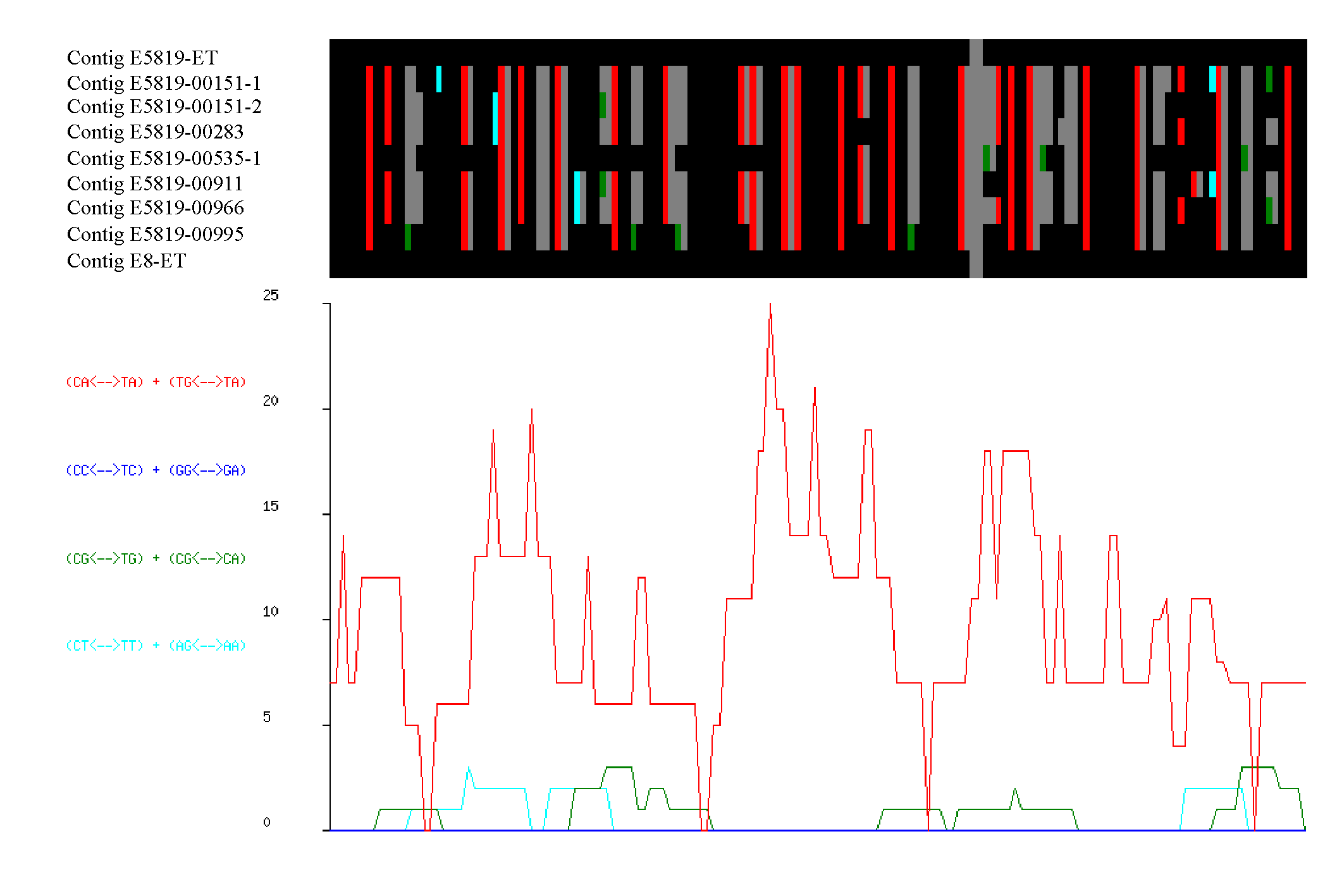


**Fig. A.1-9**. RIP in 5.8S of *Epichloë typhina*.


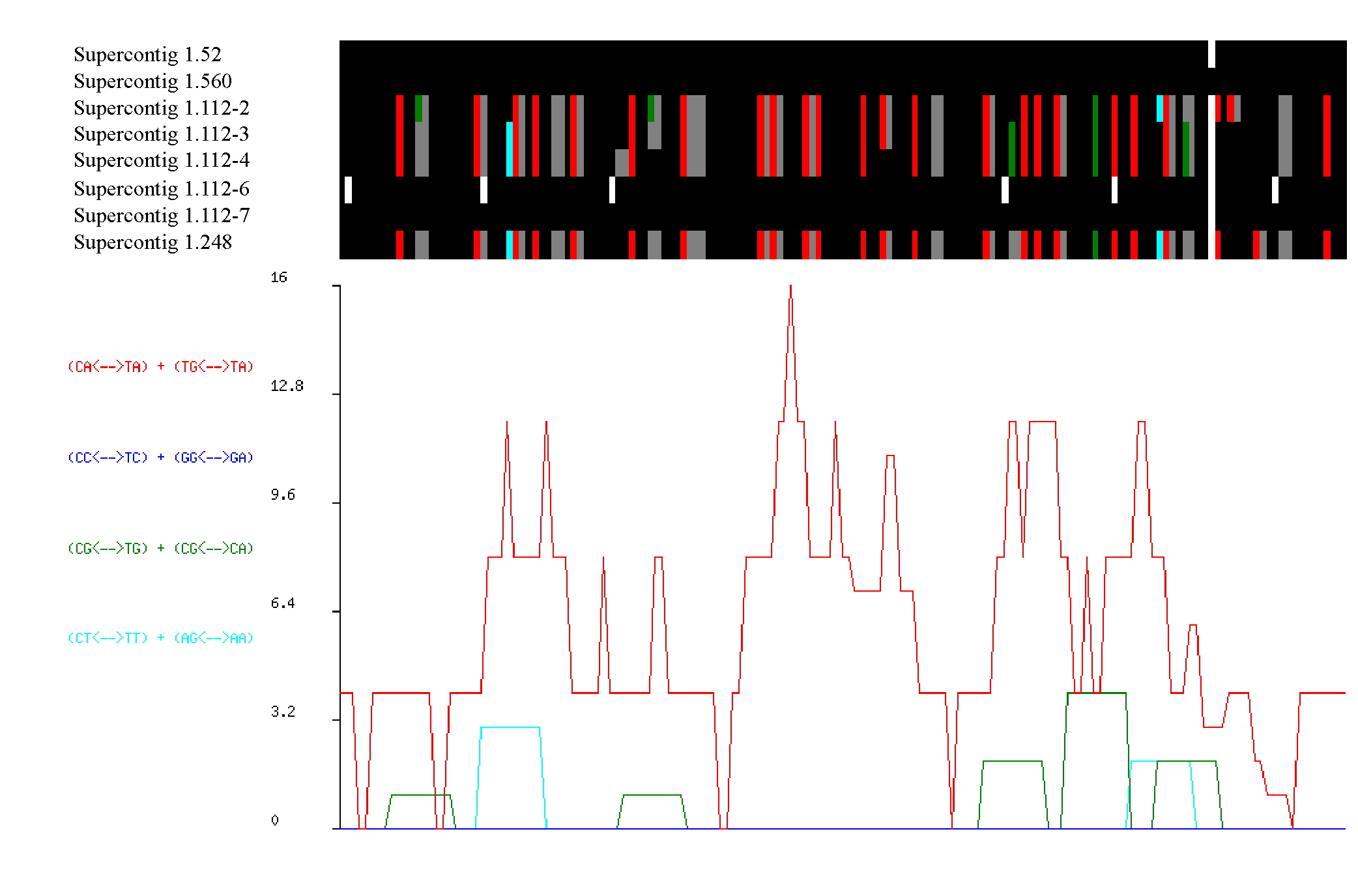


**Fig. A.1-10**. RIP in 5.8S of *Colletotrichum graminicola*.


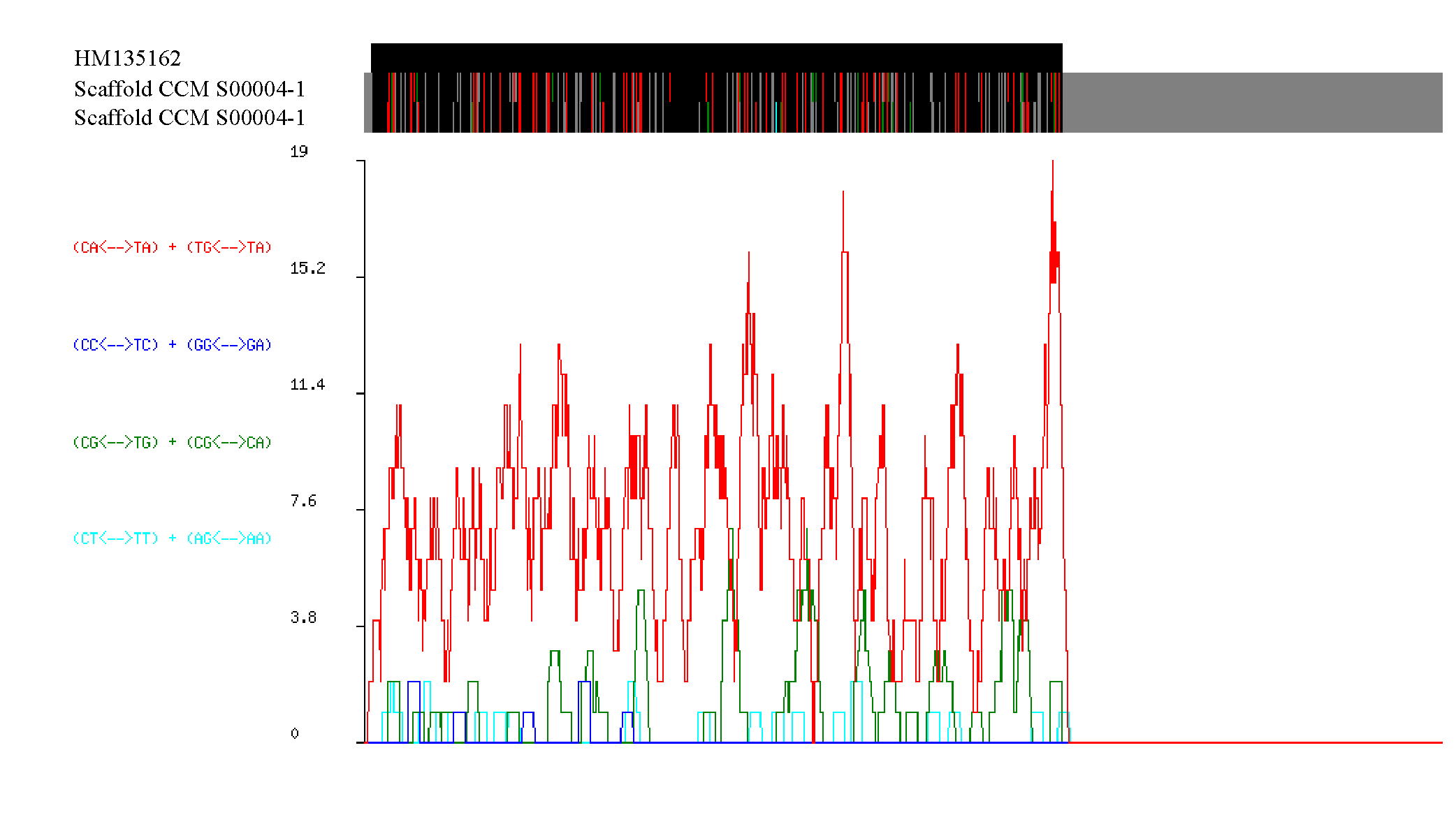


**Fig. A.1-11**. RIP in 28S of *Cordyceps militaris*.


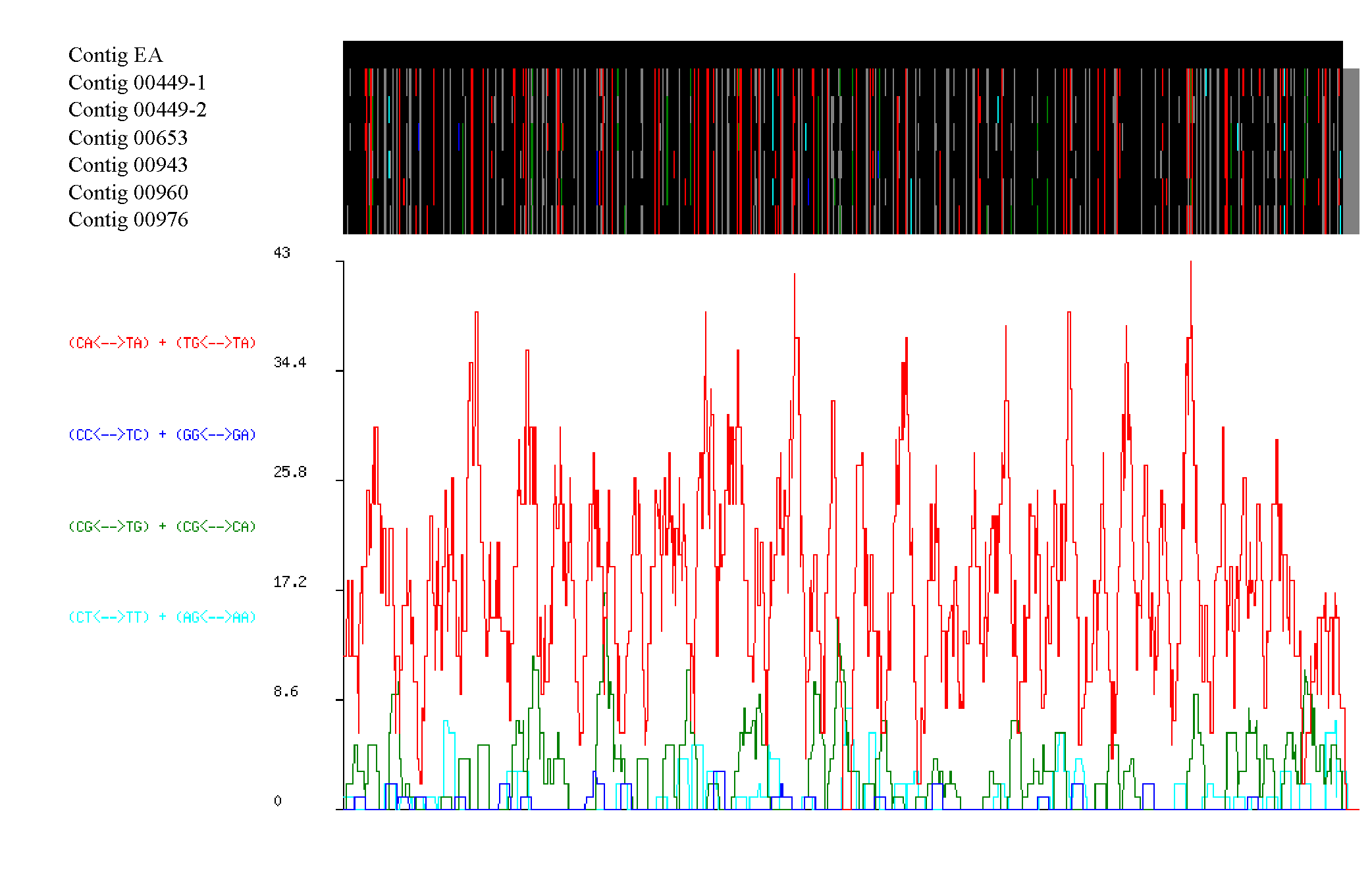


**Fig. A.1-12**. RIP in 28S of *Epichloë amarillans*.


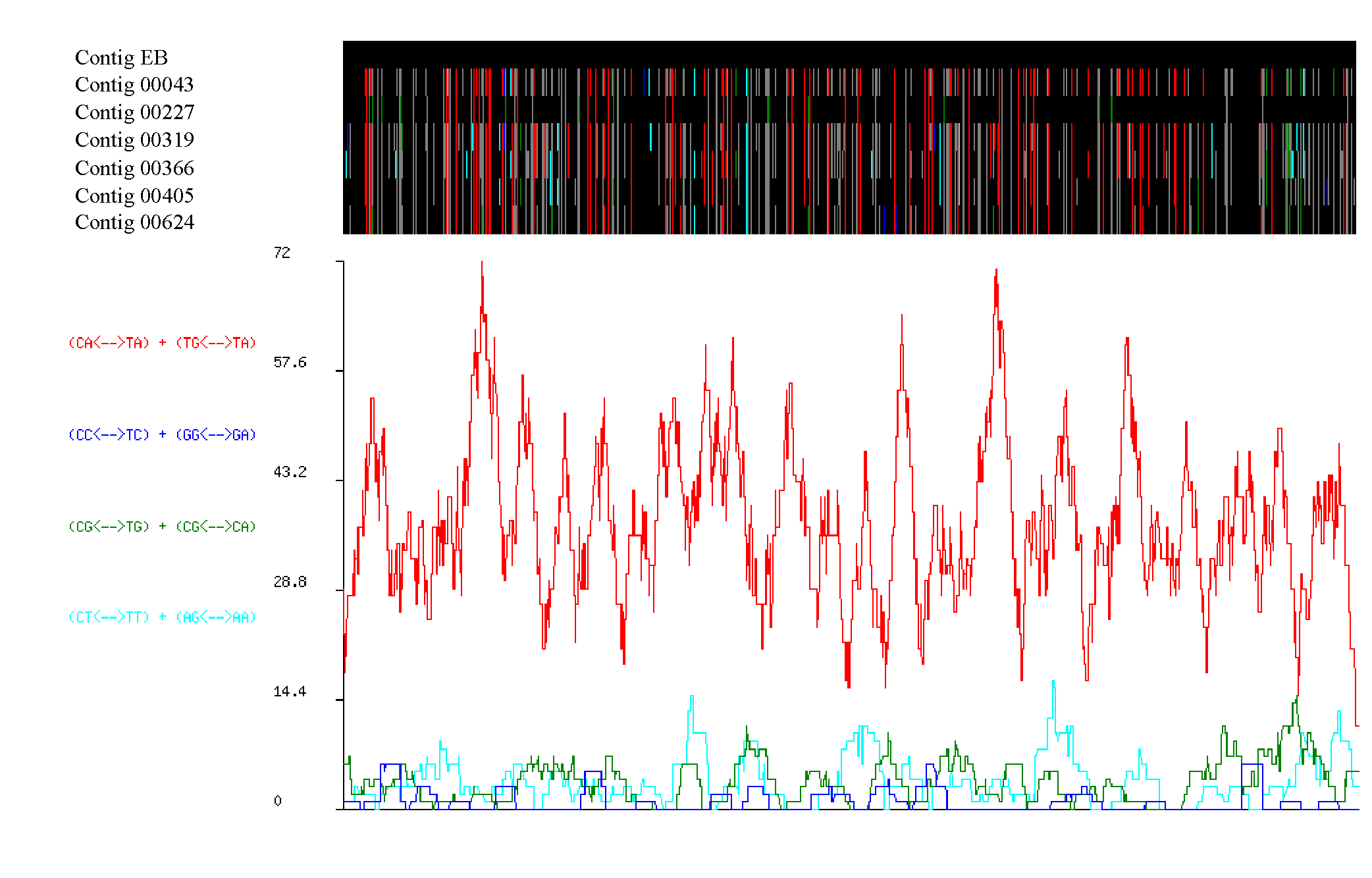


**Fig. A.1-13**. RIP in 28S of *Epichloë brachyelytri*.


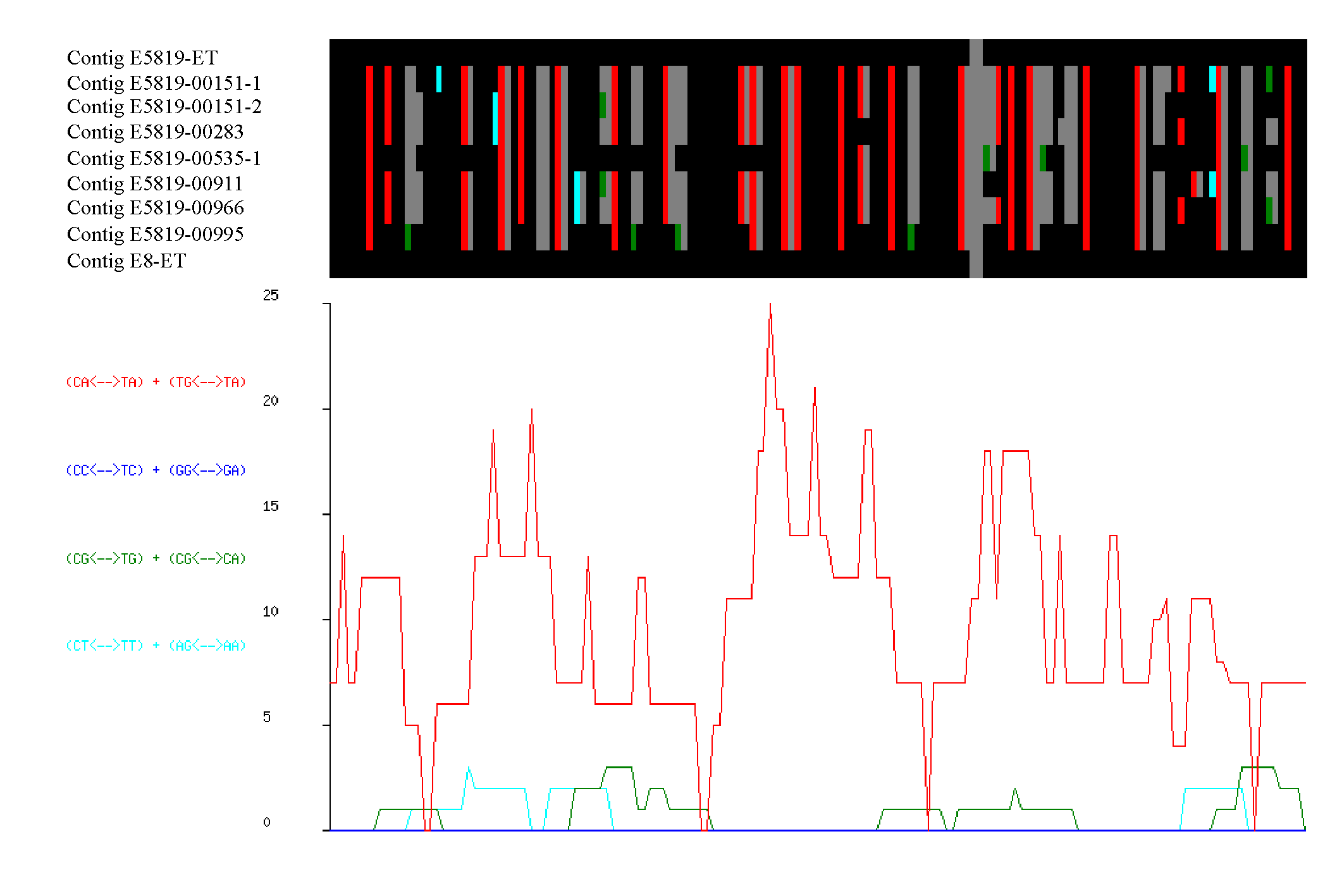


**Fig. A.1-14**. RIP in 28S of *Epichloë typhina*.


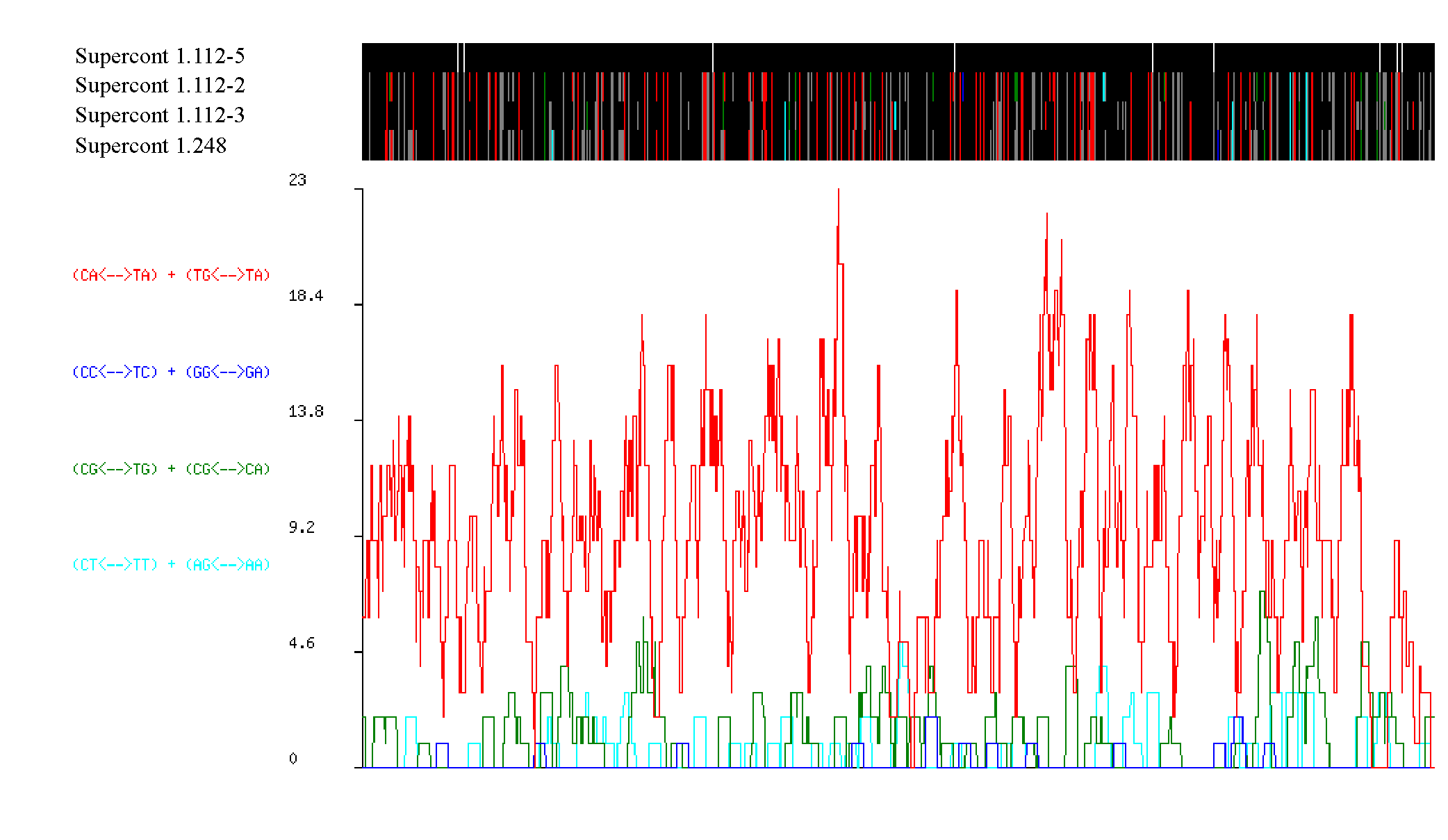


**Fig. A.1-15**. RIP in 28S of *Colletotrichum graminicola*.
